# Outcomes of Locally Advanced Rectal Cancer Patients Following Complete Clinical Response After Neoadjuvant Treatment

**DOI:** 10.7759/cureus.70432

**Published:** 2024-09-29

**Authors:** May Ashour, Sylvia Armanious, Heba Rashad, Heba Gamal, Mohamad Shalaby, Marwa Selim, Somaya Zaghloul, Eman Khorshed, Dalia Abdelfatah, Yasser Abdelazim

**Affiliations:** 1 Radiation Oncology, National Cancer Institute, Cairo, EGY; 2 Surgical Oncology, National Cancer Institute, Cairo, EGY; 3 Medical Oncology, National Cancer Institute, Cairo, EGY; 4 Pathology, National Cancer Institute, Cairo, EGY; 5 Epidemiology and Biostatistics, National Cancer Institute, Cairo, EGY

**Keywords:** complete clinical response, neoadjuvant treatment, pathological complete response, rectal cancer, watchful waiting

## Abstract

Background

Watchful waiting and non-operative management of patients with complete clinical response to neoadjuvant treatment in locally advanced rectal cancers is an emerging practice now, especially in patients whose surgery would require permanent stoma.

Methodology

This is a retrospective study of patients presenting to the National Cancer Institute, Cairo University, Egypt from January 2005 to December 2019 with pathologically proven locally advanced rectal cancer who had a complete clinical response after neoadjuvant treatment. Patients who underwent surgery after achieving complete clinical response and patients who were kept under watchful waiting were compared in terms of overall survival (OS), disease-free survival (DFS), metastasis-free survival (MFS), and local recurrence-free survival (LRFS).

Results

Of the 51 patients identified, 31 (61%) went to surgery, and 20 (39%) were kept under watchful waiting. There was no difference in five-year OS between both groups (65% for both, p = 0.57). Five-year DFS for the watchful waiting group was 68% versus 62% for the surgery group (p = 0.75). Five-year LRFS was 100% in the watchful waiting group versus 83% in the surgery group (p = 0.15).

Conclusions

Watchful waiting after a complete clinical response to neoadjuvant treatment in locally advanced rectal cancer is feasible without compromising disease-related outcomes, especially in patients with distal rectal cancers requiring permanent stoma after surgery. However, further prospective validation is needed.

## Introduction

Colorectal cancer is the seventh most common cancer in Egypt, representing 3.5% of male and 3% of female cancers [1]. Neoadjuvant fluoropyrimidine-based chemoradiation followed by total mesorectal excision (TME) with or without adjuvant chemotherapy has been a standard of care for years for locally advanced rectal cancer since the results of the German trial that reported better local control and higher sphincter preservation rates [2]. Total neoadjuvant therapy utilizing short-course radiation therapy followed by a full course of chemotherapy has also been a widely adopted protocol due to improved treatment-related outcomes [3].

Potential toxicities of neoadjuvant therapy, including pelvic radiation and surgery, are significant for patients’ quality of life, especially bowel and sexual functions in patients with low rectal tumors who would require a permanent colostomy [4-6].

This led to the idea of whether radical surgery can be avoided after complete clinical response (cCR) to the neoadjuvant treatment, given the excellent outcomes for patients with pathological complete response after surgery [7].

A growing body of literature now supports the idea of watchful waiting in clinical complete responders following neoadjuvant treatment [8-13]. The first report on this strategy was published in 2004 by Habr-Gama et al. [9] who reported excellent survival outcomes, with a five-year overall survival (OS) rate of 100% and a disease-free survival (DFS) rate of 92%. This data led us to investigate the outcomes of complete clinical responders who were kept under watchful waiting versus those who underwent surgery after achieving the same response to neoadjuvant treatment.

## Materials and methods

This is a retrospective study of patients presenting to the National Cancer Institute, Cairo University, Egypt, from January 2005 to December 2019 with pathologically proven locally advanced rectal adenocarcinoma who had a complete clinical response after neoadjuvant treatment (N = 51). Patients were eligible for neoadjuvant treatment if they were staged clinically as cT3, T4, or any T node-positive disease by magnetic resonance imaging (MRI) of the pelvis or transrectal ultrasound and if the lower edge of the tumor was within 15 cm from the anal verge. Neoadjuvant treatment included neoadjuvant radiation with or without concurrent chemotherapy (short- or protracted-course radiation therapy).

Patients who were assigned to short-course radiation therapy received a dose of 25 G over five fractions over one week. This was followed two weeks later with a full course of neoadjuvant chemotherapy (six cycles of capecitabine-oxaliplatin, CAPOX), followed by surgery after four weeks for those who went for surgery.

Patients who were assigned to protracted-course radiation therapy received a dose of 45 G over 25 fractions over five weeks to the pelvis. This was followed by a boost to gross tumor volume and mesorectum for an additional three fractions to a total dose of 50.4 G. This was concurrent with fluoropyrimidine-based chemotherapy (capecitabine/infusional Fluorouracil). Surgery was planned in six to eight weeks for those who went for surgery.

After the end of treatment, patients were restaged with an MRI of the abdomen and pelvis, colonoscopy, and multiple biopsies from the original tumor site. They were treated by either radical surgery or the watchful waiting approach.

The extent of surgery was decided at the physician’s discretion considering the original tumor location, distance from the anal verge, and post-therapy response.

Patients who were assigned to adjuvant chemotherapy started treatment within four to six weeks post-surgery. Treatment consisted of four months of fluoropyrimidine-based chemotherapy.

Patients who were offered the watchful waiting strategy were mainly those with distal rectal tumors, who would require abdominoperineal resection (APR) with a permanent stoma, or those who refused surgery.

Patients who opted for the watchful waiting approach were subjected to a strict follow-up protocol consisting of digital rectal examination, proctoscopy, and local MRI every three months during the first two years, then every six months during the third and fourth years, and then annually thereafter.

After obtaining institutional review board approval from the National Cancer Institute and obtaining patient consent, patient files were reviewed to determine treatment outcomes (frequency of clinical and pathological complete responses), patient characteristics, age, stage, histological type, surgery results, local control, and survival.

Statistical analysis

Data management and analysis were performed using SPSS version 28 (IBM Corp., Armonk, NY, USA). Numerical data were presented using means and standard deviations or medians and ranges, as appropriate. Categorical data were presented as numbers and percentages. Numerical data were explored for normality using the Kolmogrov-Smirnov test and Shapiro-Wilk test. Chi-square or Fisher’s tests were used to compare the independent groups to categorical data, as appropriate. Comparisons between two groups for normally distributed numeric variables were done using Student’s t-test. Comparisons between two groups for non-normally distributed numeric variables were done using the Mann-Whitney test.

The overall survival (OS), disease-free survival (DFS), recurrence-free survival (RFS), and metastasis-free survival (MFS) were estimated using the Kaplan and Meier method. OS was calculated from the date of diagnosis to the date of death or last follow-up. DFS was calculated from the surgery date to the date of relapse or death. RFS was calculated from the date of end of local treatment to the date of local recurrence. MFS was calculated from the date of the end of treatment to the date of distant metastasis. Differences between the survival curves were assessed with the log-rank test. All tests were two-tailed, and p-values ≤0.05 were considered significant.

## Results

Of the 51 patients identified, 31 (61%) underwent surgery, and 20 (39%) were kept under watchful waiting. The median age of the whole group was 49 years (range = 23-76). The median age in the surgery group was 46 years (range = 23-75) versus 52 years (range = 34-76) in the watchful waiting group (p = 0.027).

All patients had grade 2 adenocarcinoma. The most common site was the lower rectum in 40 (78%) patients. In seven patients, there was tumor extension to the anal canal. Further, seven (13.7%) patients had an upper rectal and four (7.8%) had mid-rectal tumors.

In this study, nine (17.6%) patients had clinically and radiologically T2 tumors, while 42 (82.4%) had T3 tumors. Patient and tumor characteristics are summarized in Table 1.

**Table 1:** Patients' and tumor characteristics P-values <0.05 are considered significant. SD: standard deviation; T: tumor; N: nodal; NA: not applicable

	Surgery	Wait & watch	Total	P-value
	n = 31(%)	n = 20(%)	N = 51(%)	
Age				
Mean ± SD	46 (23-75)	52 (34-76)	49 (23-76)	0.027
Sex				
Female	19 (61)	10 (50)	29 (57)	0.564
Male	12 (39)	10 (50)	22 (43)	
Radiological T staging				
T2	6 (19)	3 (15)	9 (18)	0.729
T3	25 (81)	17 (85)	42 (82)	
Radiological N staging				
N0	12 (39)	5 (25)	17 (33)	NA
N1	10 (32)	13 (65)	23 (45)	
N2	8 (26)	2 (10)	10 (20)	
N3	1 (3)	0 (0)	1 (2)	
Site				
Lower	23(74)	17 (85)	40 (78)	NA
Mid	3 (10)	1 (5)	4 (8)	
Upper	5 (16)	2 (10)	7 (14)	

Overall, 20 (39%) (82.4%) were kept under watchful waiting after complete clinical response to neoadjuvant treatment, with tumors located in the lower rectum for 17 (85%) patients. Moreover, 31 (61%) patients underwent surgery as the definitive local treatment, with 15 (48.3%) undergoing APR, and 16 (51.6%) undergoing low anterior resection. The entire specimen was thoroughly examined pathologically by multiple sections 5 mm apart to detect the presence of any residual disease. Nine (29%) patients had a pathological complete response, while 22 (71%) patients had microscopic residual disease. All positive nodes turned to be negative after chemoradiation.

Further, four (13%) patients developed local recurrence in the surgery group versus no recurrence in the watchful waiting group (p = 0.149). All four patients underwent low anterior resection. Two (50%) patients received a total radiation dose of 5,040 cGy, and two (50%) patients received 5,400 cGy.

Overall, four (13%) patients in the surgery group developed distant metastases, while two (10%) patients developed distant metastases in the watchful waiting group. Microscopic residual disease, despite clinical complete response in the surgery group, was seen in 22 (71%) patients, of whom 10 (45.5%) patients received short-course radiotherapy (2,500 cGy/five fractions), one (4.5%) patient received 4,500 cGy, and 11 (50%) patients received 5,040 cGy. Treatment-related details are summarized in Table 2.

**Table 2:** Treatment-related characteristics. CCRT: concurrent chemoradiation; CTH: chemotherapy; RTH: radiation therapy; IMRT: intensity-modulated radiation therapy; 2D: two-dimensional; 3D: three-dimensional; NA: not applicable

	Surgery	Wait and watch	Total	P-value
	n = 31 (%)	n = 20 (%)	N = 51 (%)	
Management				
CCRT	-	-	20 (39.2)	NA
CCRT and surgery	-	-	17 (33.3)	
CCRT and surgery and adjuvant CTH	-	-	14 (27.5)	
Adjuvant CTH				
Yes	14 (45.2)	3 (15)	17 (33.3)	0.035
No	17 (54.8)	17 (85)	34 (66.7)	
Neoadjuvant CTH				
Yes	14 (45.2)	10 (50)	24 (47.1)	0.780
No	17 (54.8)	10 (50)	27 (52.9)	
Concurrent CTH				
Yes	21 (67.7)	10 (50)	31 (60.8)	0.249
No	10 (32.3)	10 (50)	20 (39.2)	
RTH technique				
IMRT	-	-	2 (3.9)	NA
2D	-	-	19 (37.3)	
3D	-	-	30 (58.8)	
Received dose				
2,500	10 (32.3)	9 (45)	19 (37.3)	NA
4,500	1 (3.2)	0 (0)	1 (2)	
5,040	15 (48.4)	4 (20)	19 (37.3)	
5,400	5 (16.1)	5 (25)	10 (19.6)	
5,500	0 (0)	2 (10)	2 (3.9)	

**Figure 1:**
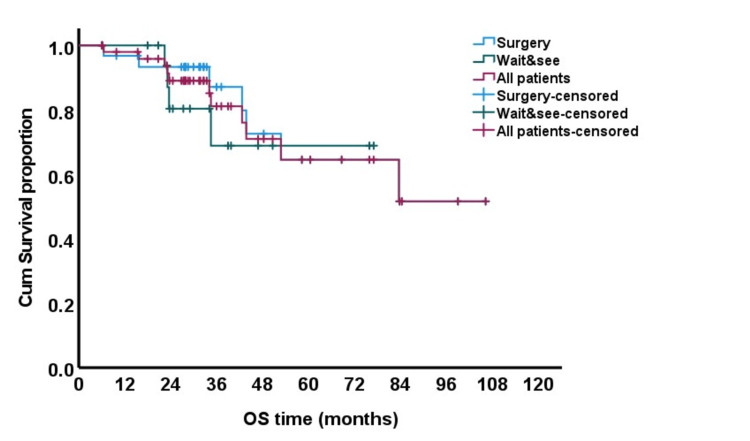
Kaplan and Meier curve for overall survival (OS).

OS at five years was 65% for the whole group, with a five-year OS of 69% in the watchful waiting group versus 65% in the surgery group (p = 0.573) (Figure 1).

**Figure 2:**
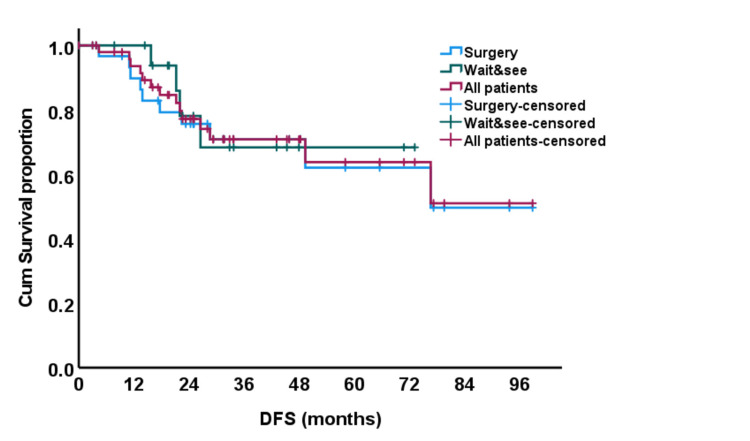
Kaplan and Meier curve for disease-free survival (DFS).

The five-year DFS for the watchful waiting group was 68% versus 62% for the surgery group (p = 0.75) (Figure 2).

**Figure 3:**
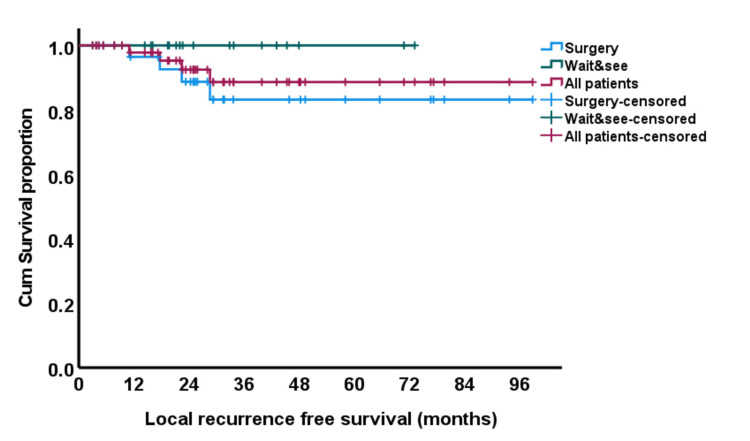
Kaplan and Meier curve for local recurrence-free survival (LRFS).

There was no statistically significant difference in terms of local recurrence-free survival (LRFS) between both groups (Figure 3). Five-year LRFS was 100% in the watchful waiting group versus 83% in the surgery group (p = 0.15).

**Figure 4:**
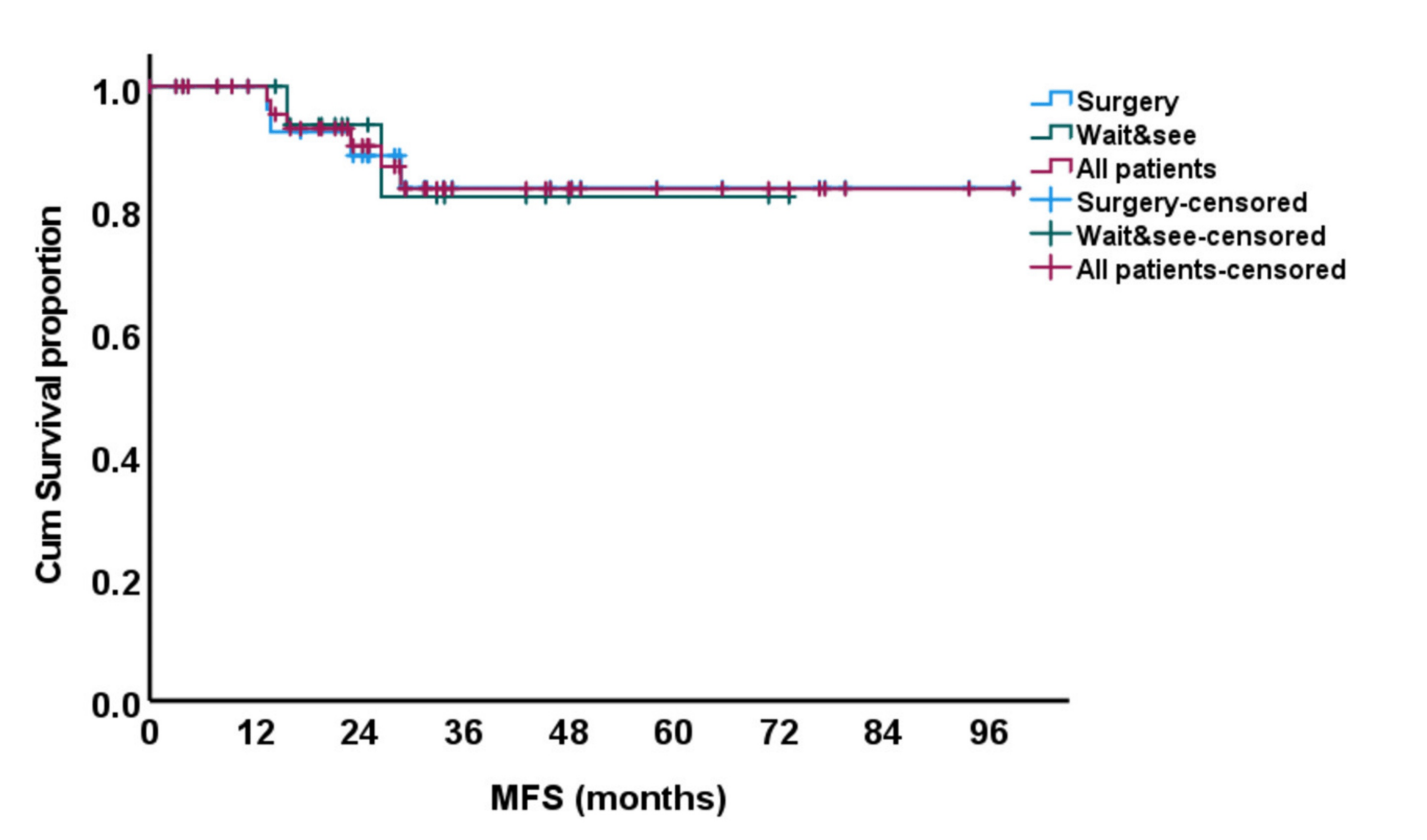
Kaplan and Meier curve for metastasis-free survival (MFS).

Furthermore, there was no difference in terms of MFS between both groups (Figure 4), with a five-year MFS of 82% in the watchful waiting group versus 84% in the surgery group (p = 0.97).

## Discussion

The watchful waiting approach in rectal cancer after a complete clinical response to neoadjuvant treatment remains an investigational approach compared to radical surgery. Oncologic outcomes in complete responders who were spared radical surgical excision remain an area of unmet research. In our study, we aimed to compare disease-related outcomes of patients who achieved complete clinical responses to neoadjuvant treatment and were kept under watchful waiting versus those who achieved the same response and underwent surgery.

Preoperative protracted-course chemoradiation in T3/4 low rectal carcinoma in a clinical trial setting demonstrated a higher incidence of pathological complete response versus short-course radiation, with pathological complete response rates of 16% and 1%, respectively [14]. In our study, the incidence of pathological complete responses in the protracted course was 29% versus no pathological complete responses in the short course.

Local regrowth occurred in 4/31 (13%) patients in the surgery group, while no local regrowth was seen in the watchful waiting group. Metastases were reported in six (11.7%) patients, with four patients in the surgery group, and two patients in the watchful waiting group. No differences in DFS and OS were seen between the watchful waiting and surgery group, which is consistent with previously published data.

Renehan et al. [11] compared the results of a watchful waiting strategy following a complete response to neoadjuvant treatment versus surgery for patients with residual disease. At three years, OS and DFS for the watch and wait group were significantly better than the surgery group at 96% and 88% for the watchful waiting group compared to 87% and 78% for surgery, respectively. Most importantly, watchful waiting was associated with significantly better colostomy-free survival (74% vs. 47%, p = 0.0001).

Smith et al. reported a local regrowth in 6/32 patients (19%), who were offered watchful waiting after a complete response to neoadjuvant treatment with all recurrences successfully salvaged by surgery [15], which is inferior to our findings.

Dossa et al. [8] reported on collective data from 23 studies including 867 patients with a local regrowth rate of 15.7% in the first two years of follow-up, with 95.4% of the recurrence successfully salvaged with surgery. There was no clinical advantage of radical surgery after achieving a complete clinical response to neoadjuvant treatment [8], which is in line with our findings.

In another report of a large international multicenter observational study including data from 880 patients who achieved a complete response to neoadjuvant treatment, with 90% of patients included in the study receiving neoadjuvant protracted-course chemoradiation [12], local regrowth occurred in 25% of patients, with 88% of recurrences in the first two years. Overall, 97% of recurrences were luminal rectal recurrences with isolated nodal recurrences rate of only 3% and a distant metastases rate of only 8%. The five-year OS was 94% for all patients and 85% for patients who experienced regrowth. These results provided a strong rationale for adopting a watchful waiting strategy for complete responders to neoadjuvant chemoradiation with disease-related outcomes better than many prospective trials using curative radical surgery as a mainstay treatment modality.

To improve the complete clinical and pathological response rates, there is a paradigm shift toward total neoadjuvant therapy to maximize treatment response, increase chances of organ preservation and non-operative management, especially in low rectal cancers, and improve disease-related outcomes. Many trials have been conducted to answer the question of the optimal sequence.

In phase II German trial CAO/ARO/AIO-12, four cycles of the chemotherapy protocol fluorouracil-leucovorin-oxaliplatin (FOLFOX) were tested either as an induction before chemoradiation or consolidation after chemoradiation, and the results showed that the consolidation arm had higher pathological complete response rates (25% vs. 17%) [16]. These results augmented the hypothesis of the longer wait after chemoradiation to improve the pathological response.

The OPRA trial was the first prospective, randomized trial conducted to answer the question of the optimal sequence of total neoadjuvant treatment [17]. Patients were randomized between neoadjuvant chemoradiation followed by consolidation chemotherapy versus the same chemotherapy used as an induction followed by chemoradiation. Clinical complete response and organ preservation rates favored the consolidation arm over the induction arm (53% vs. 41%, p = 0.01). Regrowth rates during follow-up were 27% versus 40% in favor of the consolidation arm, and, most importantly, there were no differences in DFS between patients who underwent immediate total mesorectal excision following the end of total neoadjuvant treatment and those who underwent surgery for salvage.

In summary, our data is in line with many reported data supporting non-operative management of patients who achieved clinical complete response, especially for those with distal tumors whose surgery would require permanent stoma with all its negative impact on quality of life, bowel, and sexual function, with equivalent disease-related outcomes in terms of LFRS, DFS, and OS.

Limitations of our study include the small sample size and the retrospective nature of the study with the inherent selection bias. Moreover, we did not perform quality of life assessment in patients who underwent surgery after complete clinical response.

## Conclusions

Watchful waiting and non-surgical management with a close monitoring program is not a standard-of-care approach for rectal cancer; however, data suggest that non-surgical management is a valid approach in locally advanced rectal adenocarcinoma in a select group of patients who achieve complete clinical response and whose surgery would include a permanent stoma, provided they can adhere to a strict follow-up protocol without compromise of their oncological outcomes, especially with the salvageable recurrences in most cases in published data. Better characterization and standardization of clinical complete response criteria with adequate follow-up protocols and longer follow-up is needed.
